# An unusual overrepresentation of genetic factors related to iron homeostasis in the genome of the fluorescent *Pseudomonas* sp. ABC1

**DOI:** 10.1111/1751-7915.13753

**Published:** 2021-01-25

**Authors:** Daniel Valenzuela‐Heredia, Carlos Henríquez‐Castillo, Raúl Donoso, Paris Lavín, Michael T. Ringel, Thomas Brüser, José Luis Campos

**Affiliations:** ^1^ Facultad de Ingeniería y Ciencias Universidad Adolfo Ibáñez Viña del Mar Chile; ^2^ Laboratorio de Fisiología y Genética Marina (FIGEMA) Centro de Estudios Avanzados de Zonas Áridas (CEAZA) Coquimbo Chile; ^3^ Facultad de Ciencias del Mar Universidad Católica del Norte Coquimbo Chile; ^4^ Programa Institucional de Fomento a la Investigación Desarrollo, e Innovación (PIDi) Universidad Tecnológica Metropolitana Santiago Chile; ^5^ Facultad de Ciencias del Mar y Recursos Biológicos Departamento de Biotecnología Laboratorio de Complejidad Microbiana y Ecología Funcional Instituto Antofagasta Universidad de Antofagasta Antofagasta Chile; ^6^ Network for Extreme Environments Research (NEXER) Universidad de Antofagasta Universidad de La Frontera y Universidad de Magallanes Punta Arenas Chile; ^7^ Institute of Microbiology Leibniz University Hannover Hannover Germany

## Abstract

Members of the genus *Pseudomonas* inhabit diverse environments, such as soil, water, plants and humans. The variability of habitats is reflected in the diversity of the structure and composition of their genomes. This cosmopolitan bacterial genus includes species of biotechnological, medical and environmental importance. In this study, we report on the most relevant genomic characteristics of *Pseudomonas* sp. strain ABC1, a siderophore‐producing fluorescent strain recently isolated from soil. Phylogenomic analyses revealed that this strain corresponds to a novel species forming a sister clade of the recently proposed *Pseudomonas kirkiae*. The genomic information reveals an overrepresented repertoire of mechanisms to hoard iron when compared to related strains, including a high representation of *fecI‐fecR* family genes related to iron regulation and acquisition. The genome of the *Pseudomonas* sp. ABC1 contains the genes for non‐ribosomal peptide synthetases (NRPSs) of a novel putative *Azotobacter*‐related pyoverdine‐type siderophore, a yersiniabactin‐type siderophore and an antimicrobial betalactone; the last two are found only in a limited number of *Pseudomonas* genomes. Strain ABC1 can produce siderophores in a low‐cost medium, and the supernatants from cultures of this strain promote plant growth, highlighting their biotechnological potential as a sustainable industrial microorganism.

## Graphical abstract

‘*Pseudomonas chilensis*’ strain ABC1 was fully sequenced. About 5% of the ABC1 genome is devoted to iron acquisition, including 37 ECF sigma factors. Strain ABC1 produces pyoverdine and has the genes for the production of yersiniabactin.

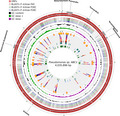

## Introduction

*Pseudomonas* is a metabolically diverse genus that thrives in multiple environments, including soil and water. Members of this genus often contain intrinsic antimicrobial resistance and/or act as opportunistic pathogens of humans, animals and plants (Silby *et al*., 2011; Jun *et al*., 2016). To colonize diverse environments, it is essential to acquire adequate amounts of iron to maintain the function of key metabolic pathways and lifestyles (Nelson *et al*., 2019). Although iron is abundant in most soils (20–40 g kg^−1^ of soil; Cornell and Schwertmann, 2003), it is often unavailable owing to its prevalence as insoluble ferric oxyhydroxide polymers under aerobic conditions (Schwertmann and Taylor, 1989). To overcome iron limitation, bacteria have evolved iron acquisition mechanisms, including the use of ferric iron‐chelating compounds called siderophores (Johnstone and Nolan, 2015).

Siderophores are strong Fe^3+^ chelators (Kf: 10^‐10^–10^‐52^ M^‐1^). There are more than five hundred known siderophores, each having unique structures and, thus, distinct proteins for synthesis, export, recognition and import (Hider and Kong, 2010). The biosynthesis of siderophores occurs *via* non‐ribosomal peptide synthetases (NRPSs) or NRPS‐independent siderophore pathways (Carroll and Moore, 2018). Recent data have also highlighted the organization of the enzymes involved in siderophore biosynthesis in multi‐enzyme complexes called siderosomes (Schalk *et al*., 2020).

In fluorescent pseudomonads, pyoverdine is the main siderophore for accessing iron (Ringel *et al*., 2016; Ringel and Brüser, 2018). Till date, almost 100 distinct pyoverdines, produced by various strains and species of fluorescent pseudomonads, have been identified (Schalk *et al*., 2020). These pyoverdines are all composed of a chromophore derived from 2,3‐diamino‐6,7‐dihydroxyquinoline linked to a peptide of 6–12 amino acids (Sandercock and Page, 2008). Pyoverdines deliver iron into the bacterial periplasm, where it is liberated. Subsequently, the iron is imported into the cytoplasm via an ABC transporter (Brillet *et al*., 2012). In addition, diverse secondary siderophores with a lower affinity for Fe^3+^ are also produced by pseudomonads, including pyochelin (PCH), pseudomonine, corrugatins and ornicorrugatins, yersiniabactin, and thioquinolobactin (Cornelis, 2010; Beaton *et al*., 2018). The siderophore biosynthesis is finely regulated by the availability of iron and diverse transcriptional regulators such as the ferric uptake regulator (Fur) or extracytoplasmic function sigma factors (EFCsf) PvdS and FpvI (Bonneau et al., 2000; Ringel and Brüser, 2018; Schalk *et al*., 2020).

Fur is a transcriptional repressor that acts both directly and indirectly through several signal transduction pathways to handle iron absorption (Leoni *et al*., 2000). Fur binds iron and attaches itself to a consensus sequence (Fur box) in the promoter region of iron‐regulated genes (Marcoleta *et al*., 2018). In the presence of iron, Fur inhibits iron conservation strategies by suppressing the production of two key small ribonucleic acids (sRNAs): PrrF1 and PrrF2 (Sonnleitner *et al*., 2010). Conversely, in the absence of iron, these small RNAs are synthesized and facilitate the inhibition of genes that encode ‘non‐essential’ iron‐containing proteins (Chareyre et al., 2018). Recently, it has been reported that ErsA, a novel sRNA characterized in *P. aeruginosa,* also responds to limited iron availability (Falcone *et al*., 2018).

ECFsf are multi‐domain subunits of the bacterial RNA polymerase that play critical roles in transcription initiation (Otero‐Asman *et al*., 2019). Frequently, iron starvation gene clusters contain ECFsf, which are co‐transcribed in conjunction with a transmembrane anti‐sigma factor that keeps them inactive (Moraleda‐Muñoz *et al*., 2019). In this case, activation only occurs in response to a specific signal through a transduction pathway involving an outer membrane protein belonging to a TonB‐dependent receptor family that regulates uptake of a siderophore or haeme group (Ye *et al*., 2014). This signal transduction system is termed cell surface signalling (CSS; Bastiaansen *et al*., 2017). In *P. aeruginosa*, ECFs related to iron starvation integrate a complex regulatory network for regulation of iron homeostasis (Chevalier *et al*., 2019).

In this work, we demonstrate that *Pseudomonas* sp. strain ABC1 is a representative of a novel species tentatively named ‘*Pseudomonas chilensis’* strain ABC1 (Valenzuela‐Heredia *et al*., 2020), which forms a sister clade of the recently proposed *Pseudomonas kirkiae*. Comparative genomics revealed an overrepresentation of genes related to iron metabolism in both strains, specifically for iron homeostasis and transport. Particularly, the strain ABC1 has a putative NRPS system for production of *Azotobacter*‐type pyoverdine, which differs from the *Pseudomonas*‐type, and a hybrid NRPS/T1PKS system for synthesis of an additional siderophore similar to yersiniabactin but rarely assigned to the genus *Pseudomonas* (Jones *et al*., 2007; Beaton *et al*., 2018). Remarkably, supernatants containing siderophores from the ABC1 strain promoted growth of the model plant species *Arabidopsis thaliana*. Therefore, strain ABC1 exhibits promising characteristics for application in biotechnology, plant crops and synthetic biology.

## Experimental procedures

### Bacterial isolation and identification

The strain ABC1 was isolated from soil interstitial water in Valparaiso, Chile (33.035021 S 71.595079 W) by a liquid pre‐culture on acetate, ammonia and mineral media (AAM; Jungles *et al*., 2011). This was followed by single colony passages onto AAM agar, each one incubated for 72 h at 20°C. The strain was deposited at the Chilean collection of microbial genetic resources (CChRGM), Chillan, Chile, with accession number RGM 2961.

### PacBio sequencing of the Pseudomonas sp. ABC1

A single colony of the ABC1 strain was processed by Macrogen Inc. Korea and sequenced using the PacBio RSII platform. The sequencing of the ABC1 strain produced a unique contig of 4,035,896 bases with a mean coverage of 209x (Valenzuela‐Heredia *et al*., 2020). The raw sequence assembly is available under the BioProject accession number PRJNA641577.

### Pseudomonas sp. ABC1 taxonomy and genome comparison

The taxonomic inference of the ABC1 strain was performed with a multilocus sequence analysis (MLSA), with concatenated partial sequences of the genes encoding the 16S ribosomal RNA (rRNA), DNA gyrase B (*gyrB*), RNA polymerase sigma factor (*rpoD*) and RNA polymerase beta subunit (*rpoB*; Gomila *et al*., 2015). Sequences were aligned using MAFFT v7, using the L‐INS‐i option with genes from related *Pseudomonas* genomes. A phylogenetic tree was constructed using IQ‐TREE (Nguyen *et al*., 2015) with the ‐m TEST, ‐bb 1000, ‐alrt 1000 options. Sequences are available under GenBank accession numbers MW029818, MW030956, MW030957 and MW030958.

Genomes from the genera *Pseudomonas* and *Azotobacter* (40) were downloaded from the National Center for Biotechnology Information (NCBI) and analysed together using anvio v5 (Eren *et al*., 2015; see Table S1). The whole‐genome average nucleotide identity (ANI) was calculated with FastANI (Jain *et al*., 2018). In silico DNA–DNA hybridization (isDDH) was calculated with the genome‐to‐genome distance calculator (GGDC; Meier‐Kolthoff *et al*., 2013). A phylogenetic tree was generated using 137 single‐copy genes reported in Campbell *et al*. (2013). Sequences were aligned with MAFFTv7 (Katoh & Standley, 2013), using the L‐INS‐i option. The phylogenetic tree was inferred from the concatenated alignment of the single‐copy genes using IQ‐TREE with the ‐m TEST, ‐bb 1000, ‐alrt 1000 options. A cluster of the pangenome was generated with the Anvio software based on the presence/absence of genes in each genome using the Markov clustering (MCL) algorithm (inflation = 10) through Euclidean distance and wardD2 linkage.

### Functional features of the Pseudomonas sp. ABC1

Annotation of predicted genes was performed using Diamond (Buchfink *et al*., 2015), with the sensitive option to find homologues in different databases, including the Kyoto Encyclopedia of Genes and Genomes (KEGG), Pfam, the Clusters of Orthologous Groups (COGs) and FeGenie (Garber *et al*., 2020). Distribution of genes, COG categories, KEGG orthology and Pfam modules across genomes was analysed in the R environment. Furthermore, the ABC1 genome was analysed through the MicroScope Microbial Genome Annotation and Analysis Platform (Vallenet *et al*., 2020) and antiSMASH v5.1.2 (Blin *et al*., 2019).

### Siderophore production

The production of siderophore by *Pseudomonas* sp. ABC1 was first confirmed by the Chrome Azurol S (CAS) assay (Payne, 1994). For bioreactor siderophore production, *Pseudomonas* sp. ABC1 was grown under batch fermentation in 5 l biological reactors with AAM medium. Batch culture was performed at 20ºC. The pH was measured with a pH meter (Oakton, WD‐35613) at the initial and end stage. Siderophore production was tested in aerated and non‐aerated conditions. For aerated conditions, 1 vvm of air was supplied by an air pump (Sera, 550R). The supernatant from each condition was then filtered through a filter with 0.2 µm pore size. Siderophore concentration was quantified spectrophotometrically using the FeCl_3_ titration method (Jurkevitch *et al*., 1986).

To evaluate the interaction of siderophores with metal ions, 10 ml of 0.2 µm filtered supernatants with a concentration of 20 mg l^‐1^ of siderophores were exposed to 1 ml of 0.5 mM solutions of Fe^3+^, Mn^2+^, Cu^2+^, Co^2+^ or Zn^2+^ (see Table S2). We evaluated the fluorescence of the siderophore by exposing the filtered supernatants to UV light with a transilluminator.

### Siderophore structure prediction

Initially, NRPSs potentially involved in pyoverdine biosynthesis (see Fig. 3 and Table S3) and their respective A‐domain substrate specificities were predicted using antiSMASH v5.1.2. Different domains in the NRPS‐PKS were also identified using PKS/NRPS Analysis website (Bachmann and Ravel, 2009). However, substrate specificities could only be assigned to a limited number of A‐domains. To predict the structure of the unknown pyoverdine, further bioinformatic analyses were necessary. First, known pyoverdine and azotobactin structures were collected from literature and, when possible, genomic data were downloaded from the NCBI database or the *Pseudomonas* genome database (Winsor *et al*., 2016). Subsequently, the collected genomes were analysed using antiSMASH v5.1.2. From the known structures of pyoverdines and azotobactins, A‐domain substrate specificities were inferred and assigned to the respective domains. Thereafter, the specificity‐conferring Stachelhaus and Angstrom codes (Stachelhaus *et al*., 1999; Röttig *et al*., 2011) were extracted from all newly annotated A‐domains and collected in a database. By comparing the specificity‐conferring codes of the unassigned A‐domains with the annotated codes collected in the database, a likely structure was deduced. The process described above was partially automated using in‐house developed Python scripts.

### Siderophore phytostimulation in Arabidopsis thaliana

Surface sterilized *A. thaliana* (Col‐0 ecotype) seeds were stratified at 4°C for 2 days. Then, seeds were sown in a sterile agar‐solidified Hoagland medium (5 mM KNO_3_, 5 mM Ca(NO_3_)_2_, 2 mM MgSO_4_, 50 µM H_3_BO_3_, 2 µM KI, 15 µM ZnSO_4_·7(H_2_O), 3 µM Na_2_MoO_4_·2(H_2_O), 5 µM MnSO_4_, 50 nM CoCl_2_·7(H_2_O), 50 nM CuSO_4_·5(H_2_O), pH 6). Twenty‐four sown seeds were irrigated with 0.5 mL of supernatants filtered with a concentration of 20 mg L^‐1^ of siderophores from strain ABC1, and 24 seeds were irrigated with 0.5 mL sterile water. Agar plates were placed vertically in a growth chamber with white light (under cycle of 12‐12 dark–light at 20°C). Under sterile conditions, six germinated plants were irrigated with 0.5 ml of supernatants filtered with a concentration of 20 mg l^‐1^ of siderophores from strain ABC1, and six plants were irrigated once with 0.5 ml sterile water. This set was incubated for 8 days with white light (under cycle of 12‐12 dark–light at 20°C). To measure the foliar area, pictures of these plants were analysed with the ImageJ software. Data were statistically analysed by unpaired *t*‐test.

## Results and Discussions

### Taxonomic affiliation of Pseudomonas sp. ABC1

**Fig. 1:**
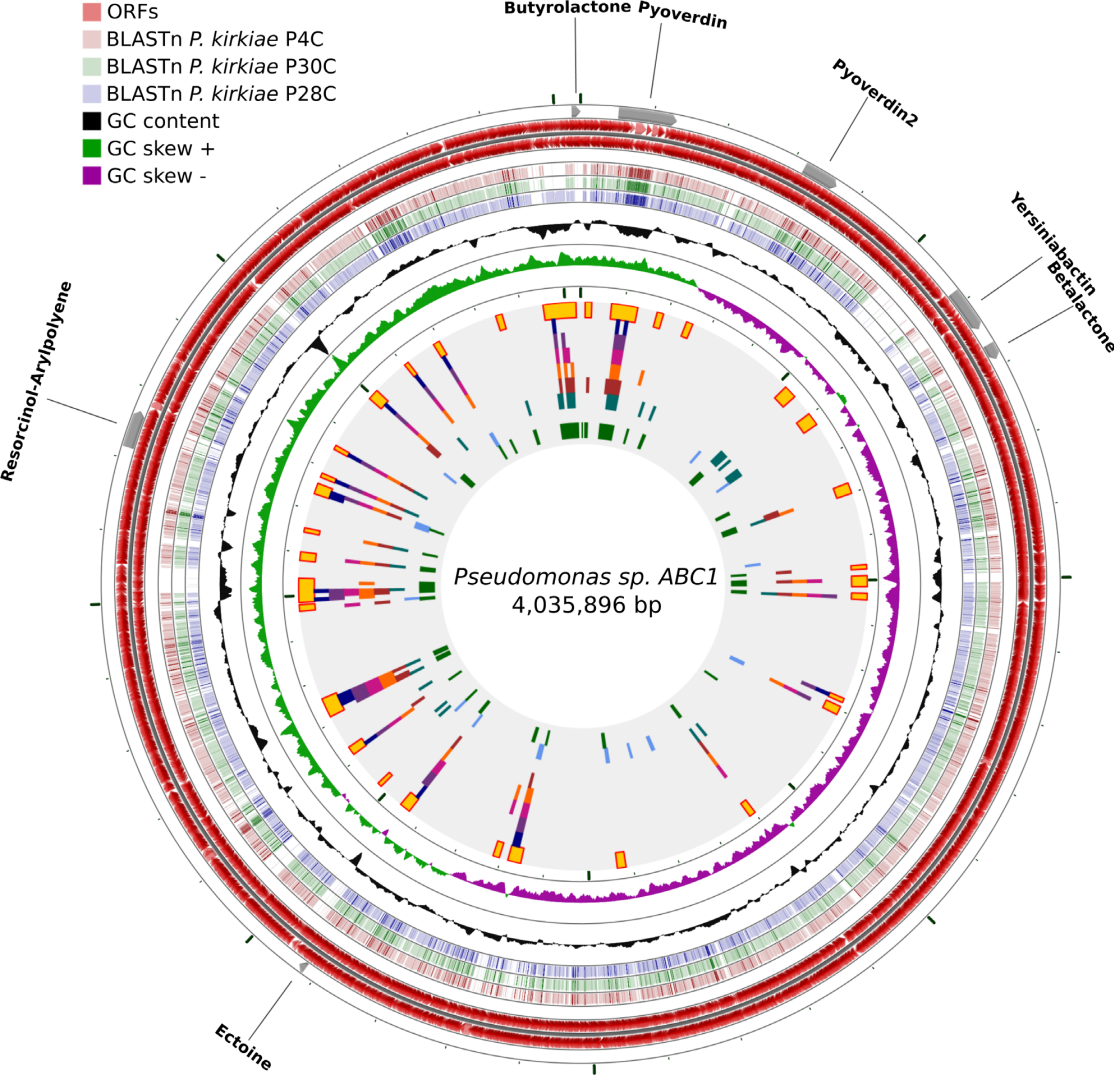
CGView map of the *Pseudomonas* sp. ABC1 genomic features. Starting from the outermost ring, the feature rings depict antiSMASH prediction of secondary metabolites, forward‐strand sequence features and reverse‐strand sequence features. The next three rings show regions of sequence similarity detected by BLASTn comparisons conducted between CDS translations from the ‘*P. chilensis’* ABC1 and three *P. kirkiae* comparison genomes. Then, the next three rings display the GC content and GC skew (+ and ‐). Finally, the inner rings correspond to the potential genomic islands detected based on GC content, dinucleotide and tetranucleotide frequency, and K‐mers distribution (2,3,4,5,6) respectively. Yellow squares correspond to regions detected more than once.

The recently sequenced *Pseudomonas* sp. ABC1 (see Fig. 1; Valenzuela‐Heredia *et al*., 2020) appears to be related to the *P. kirkiae* clade isolated from oak in the United Kingdom (Bueno‐Gonzalez *et al*., 2020). MLSA analysis showed that strain ABC1 has a nucleotide sequence identity of 94% to *P. kirkiae* strain 4C (see Fig. S1). The ANI between the ABC1 and *kirkiae* strains was ~ 86.2% (see Fig. S2), with isDDH values of 45.7–47 % and a difference of 1.44 to 1.37% in G + C. Genome similarity values of strain ABC1 and related *P*. *kirkiae* strains are well below the accepted thresholds of 95 % ANI and 70 % isDDH for species delineation and provide support for the classification of a novel species, which we tentatively termed as ‘*P. chilensis’*, with its type strain being ABC1.

### Comparative genomics and phylogenomic analysis of Pseudomonas sp. ABC1

Regarding functional representation, from the 3,483 genes present in the ABC1 genome, 2,822 predicted KEGG orthology (see Table S4). The top five represented KEGG modules corresponded to the aminoacyl‐tRNA biosynthesis (M00359), citrate cycle (M00009), ribosome (M00178), iron complex transport system (M00240) and peptides/nickel transport system (M00239). A total of 1642 predicted KOs were shared across analysed genomes, corresponding to 80% of the predicted KOs for ABC1, 73% for *P. kirkiae* and 57.7% for *Azotobacter* (see Fig. S3a), with only five KOs being unique to the ABC1 (see Table S4). These genes were located in non‐ribosomal clusters or genomic islands and are present in few *Pseudomonas* genomes from distant clades not included in the pangenomic comparison.

Based on the COG functional annotation, 63 genes with a known function appeared unique to the strain ABC1 (see Fig. S3b). Most of these genes corresponded to the following COG categories: Inorganic ion transport and metabolism (P; 31.4%), signal transduction mechanisms related to inorganic ion transport and metabolism (P|T; 7.14%), and transcription (K; 8.6%). Specifically, functions related to NRPS, iron complexes, iron siderophore transport systems and iron‐related regulatory proteins were among the unique genes detected for ABC1 strain (see Table S4).

Overall, *Pseudomonas* sp. ABC1 has the genetic imprints for intensive iron scavenging and homeostasis. In this genome, the iron complex transport systems appear as the top represented functional module; conversely, in *P.kirkiae* and *P. stutzeri*, the peptides/nickel transport and the CheA‐CheYBV (chemotaxis) two‐component regulatory system were the top represented functional modules respectively. This reflects the differences in the genomic potential in related *Pseudomonas* strains.

### Genomic determinants to iron

**Fig. 2:**
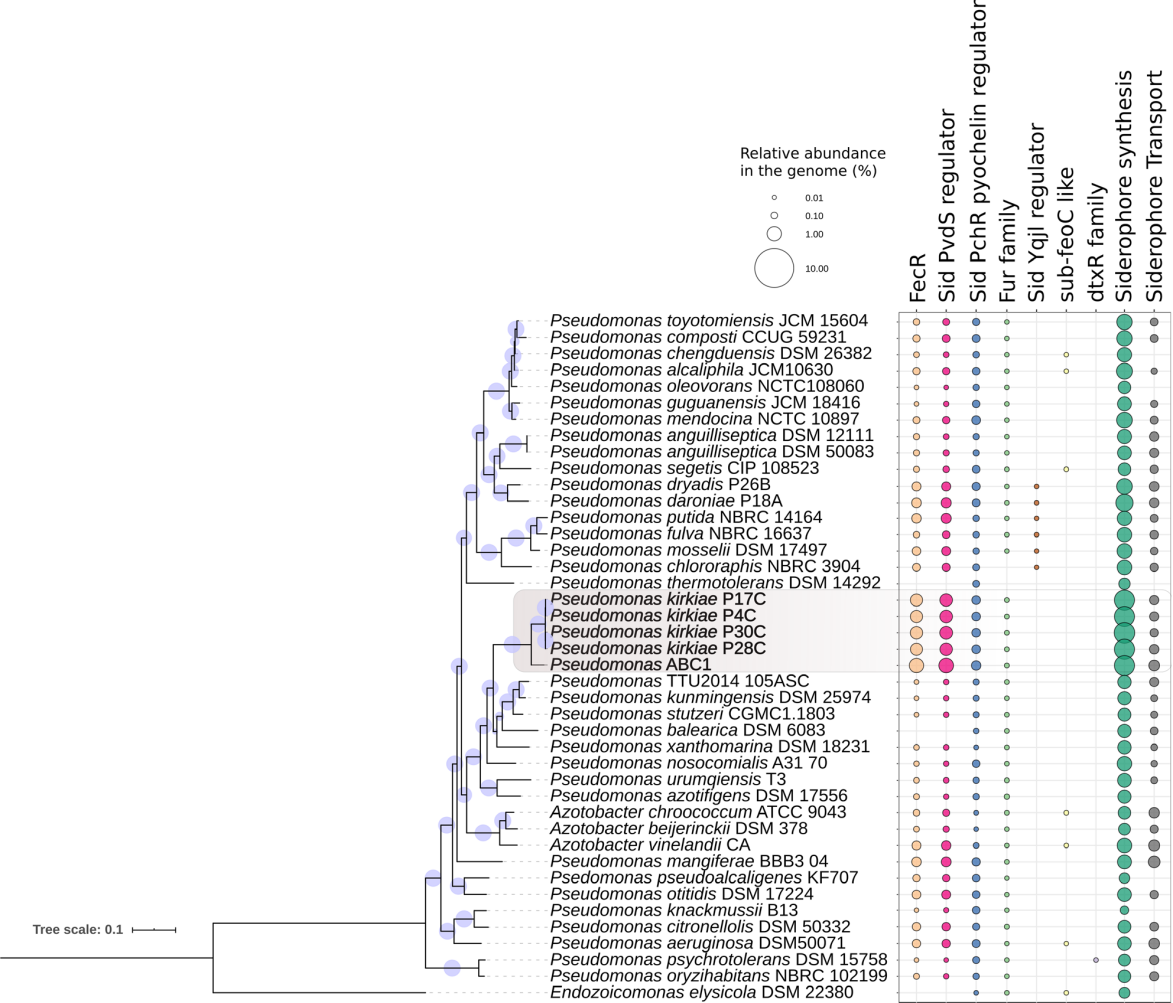
ML tree reconstruction and iron‐related genes of the *Pseudomonas* sp. ABC1 and related strains. The ML tree reconstruction was based on multiple concatenated marker genes (139) selected in Anvio. Amino acidic sequences were aligned with MAFFT using the L‐INS‐i option. The ML topology exhibited SH‐like approximate likelihood ratio support values (*n* = 1000) at each node (values > 50% are shown), and the model selected was LG + F+I + G4. Model selection and tree reconstruction was performed with IQ‐TREE2. Iron‐related genes were detected using FeGenie. Each iron‐related function corresponds to the percentage of the total coding sequences in each genome.

A total of 195 gene clusters related to iron utilization were found in the ABC1 strain, corresponding to 5% of its total CDS, which is higher than the observed percentage in *P.kirkiae* genomes (~4.65 %). However, in both ‘*P. chilensis*’ ABC1 and *P.kirkiae* genes, iron homeostasis was overrepresented when compared to related species (see Fig. 2). Most of these genes in ABC1 corresponded to iron gene regulation (*n* = 84) and acquisition (*n* = 79), including the uptake of xenosiderophores. Remarkably, we identified an overrepresentation of ECFsf related to iron starvation across the genome (37 genes), with 36 clusters involved in the activation of TonB‐dependent transporters (see Fig. S4 and Table S4).

**Fig. 3:**
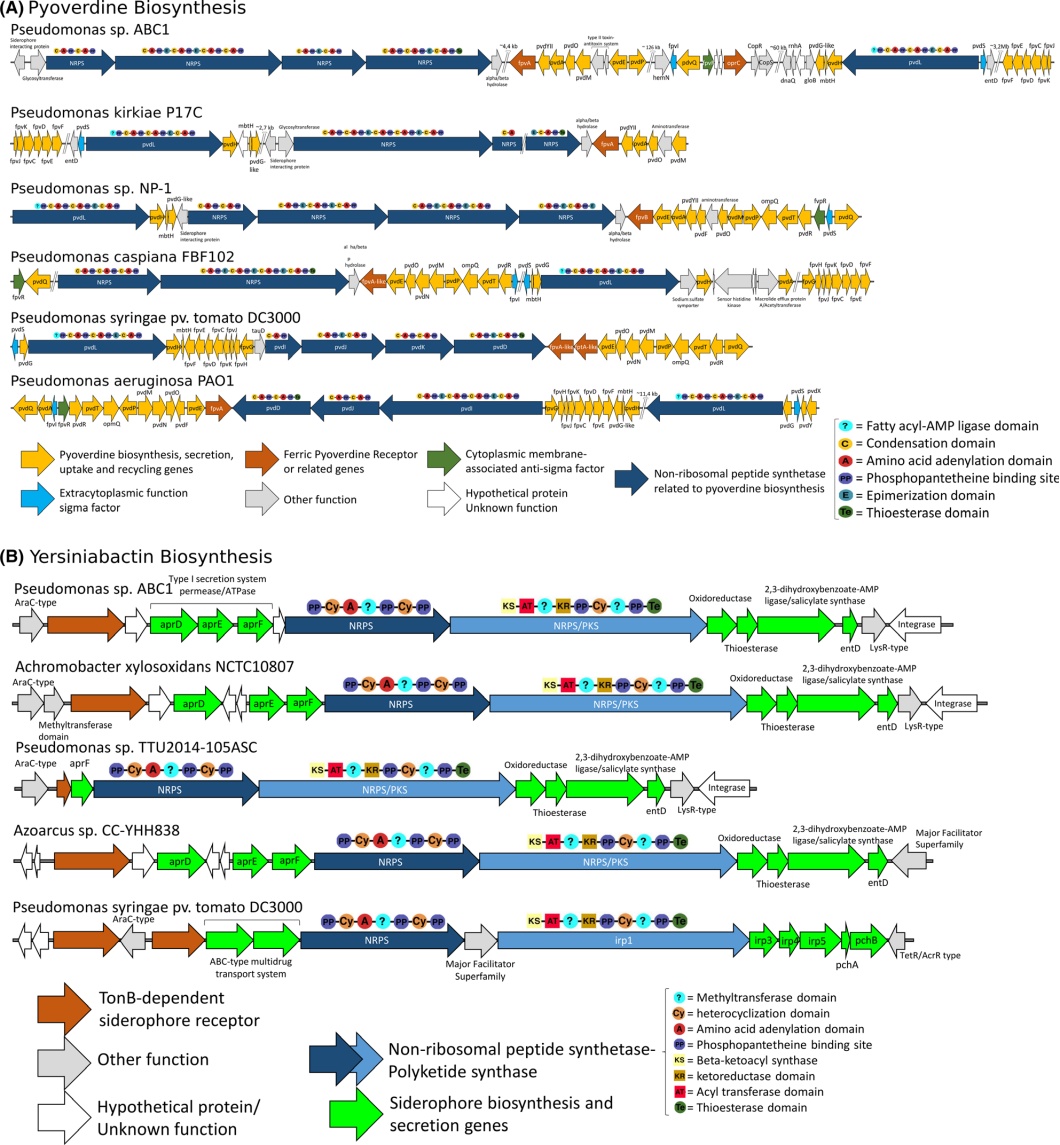
Genomic content of the genes involved in the NRPS of pyoverdine and yersiniabactin present in *Pseudomonas* sp. ABC1 and related genomes. Genomes were selected according to BLASTp homology to the non‐redundant protein sequences (nr) database in NCBI. The *Pseudomonas syringae pv*. *tomato* DC3000 was selected as a reference siderophore‐producing strain. A. Genomic context of the pyoverdin related genes. B. Genomic context of the yersiniabactin producing genes. Gene clusters were obtained based on antiSMASH identification of secondary metabolites biosynthetic genes. Genes were coloured according to function. Different domains in the NRPS‐PKS were denoted in coloured symbols.

Regarding ECFsf, we found the gene coding not only for PvdS and FpvI but also for a FpvR‐like anti‐sigma protein inside the pyoverdine biosynthesis cluster (see Fig. 3), suggesting canonical mechanisms for the regulation of the siderophore production. In addition, two additional ECFsf genes with unknown functions clustered together with *pvdS* (see Fig. S4). In addition, 24 ECFsf phylogenetically related to *foxI/fiuI/femI/fecI/pupI* genes were also found (see Fig. S4). Those genes are involved in xenosiderophores acquisition in *P. aeruginosa* and *P. putida* (Koster *et al*., 1994; Banin *et al*., 2005; Llamas *et al*., 2006, 2008). Additionally, strain ABC1 harbours the *hasI* gene, its gene context (*has* genes) related to haeme acquisition and an extra *hasI*‐like gene (see Fig. S4), suggesting that ABC1 has more than one system for haeme acquisition. This strain also contains five extra ECFsf related to *fpvI* (pyoverdine acquisition) and *hxuI* (haeme sensing) genes from *P. aeruginosa* and two other ECFsf phylogenetically distant (see Fig. S4 and Table S4). Overall, these results suggest a high number and diversity of CSS systems dedicated to iron in this strain, including CSSs that are absent in other *Pseudomonas* species.

Regarding the regulation of iron homeostasis, 11 putative Fur‐binding sites were found in promoter regions of genes related to FecI‐family ECFsf and their FecR‐type regulatory interaction partners in *Pseudomonas* sp. ABC1 (see Fig. S4). Additionally, other 26 sequences predicted to be recognized by Fur were also found (see Table S4). Moreover, the ABC1 strain contains a single *prrF* gene of 145 bp, similar to what was found in *P. stutzeri*, *P. xanthomarina* and *Azotobacter vinelandii*; this differs from other *Pseudomonas* species that contain two *prrF* genes, as reported by Reinhart *et al*. (2017). Finally, in ABC1, an *ersA*‐like sequence was found located in an ECFsf cluster involved in iron homeostasis (see Fig. S4 and Table S4).

To avoid intracellular iron toxicity, the putative cation diffusion facilitator (CDF) encoded by the *aitP* gene likely mediates iron efflux in strain ABC1 (Salusso and Raimunda, 2017; see Table S4). Furthermore, intracellular iron can be stored by two bacterioferritins encoded by the *bfrB* and *ftnA* genes (see Table S4). The *ftnA* coding sequence is located adjacent to an ECFsf cluster involved in iron homeostasis (see Fig. S4), while in *P. kirkiae* the only adjacent gene from this cluster is a *fec*A‐like gene. We only found one catalase encoded by *katG* (Sandercock and Page, 2008), three superoxide dismutases (SOD), an Mn‐SOD encoded by *sodA*, an Fe‐SOD encoded by *sodB,* and an Cu‐Zn SOD (Martins *et al*., 2018). In addition, two cytochrome *c_551_* peroxidase copies were found in the ABC1 genome, with one of them being associated with an ECFsf cluster involved in iron homeostasis (see Fig. S4 and Table S4).

Taken together, these results show the complex and intricate molecular mechanisms involved in iron regulation. The association between ROS control and ECFsf clusters also highlights a possible coordination to avoid stress during iron internalization and storage. This coordination has been described in terms of gene expression for *P. aeruginosa* when acetate is the sole source of carbon (Ha *et al*., 2018).

### Metabolic gene clusters in Pseudomonas sp. ABC1

The genome of ABC1 strain has seven metabolic clusters (see Fig. 1) that harbour 199 biosynthetic genes (see Table S3). Two clusters correspond to NRPS with lower similarity to pyoverdine biosynthetic clusters (with 9 and 4% of similarity; see Fig. 3A). The hybrid NRPS/T1PKS cluster 3 shows a low similarity (8%) with the NRPS‐PKS for the siderophore yersiniabactin (see Fig. 3B). The betalactone cluster 4 has no similarities with known clusters in databases. The ectoine cluster 5 presents a high similarity to known ectoine clusters (50%). The hybrid resorcinol/aryl polyene cluster 6 also presents similarity with known clusters of APE Vf (45%). Cluster 7 for butyrolactone has no similarity to known clusters in databases (see Table S3).

Most of these clusters displayed a gene configuration similar to that of *P*. *kirkiae* strains. However, in *P. kirkiae,* these clusters appear incomplete or even disrupted, possibly due to the partial completeness of their genomes (see Fig. 3A and Table S3). Also, in strain ABC1, most of the secondary metabolite clusters are located in zones of the genome characterized as genomic islands (see Fig. 1). The presence of hybrid genes in strain ABC1 (i.e. yersiniabactin and butyrolactone clusters) common to *Pseudomonas* from distant clades and species (Table S3) suggests a frequent horizontal as well as vertical gene transfer in these clusters (Bultreys *et al*., 2006; Fischbach *et al*., 2007; Aiman *et al*., 2018). These proteins play important roles in the ecology and physiology of microorganisms, mediating interactions both among microbial species and between microbes and multicellular organisms (Fischbach *et al*., 2007); moreover, these are relevant to biomedical and biotechnological research. Butyrolactone is a key molecule for sensing quorum, promoting antibiotic production, and having effects towards *Pectobacterium carotovorum* (formerly *Erwinia carotovora*) and phytopathogenic fungus *Botrytis cinerea* (Cazar *et al*., 2005; Biarnes‐Carrera *et al*., 2015; Theobald *et al*., 2019). Signalling circuits based on quorum sensing mechanisms have been popular tools for synthetic biology, and the expression of biosynthetic genes in a plug‐and‐play fashion is excellent candidates to programme cells to generate complex functions (Lee and Zhang, 2006, 2015; Theobald *et al*., 2019).

### Pyoverdine biosynthetic cluster and structure prediction

Five large NRPS genes were found in strain ABC1 genome (see Fig. 3 and Tables S4, S3), which are related to *pvdD*, *pvdI*, *pvdJ* and *pvdL* from *Pseudomonas aeruginosa* involved in the biosynthesis of the pyoverdine precursor peptide (Imperi and Visca, 2013; Gasser *et al*., 2020; see Fig. 3 and Table S4). Additionally, we detected *pvdH, pvdA and mbtH* genes; this last encodes a protein associated with the NRPS of pyoverdine synthesis that can enhance the solubility or activity of the NRPS of the siderophore in *P*. *aeruginosa* (Miller *et al*., 2016). Interestingly, the soluble thioesterase PvdG that provides functionality *in trans* for NRPSs is absent in ABC1. However, this strain encodes a thioesterase in the pyoverdine cluster (see Fig. 3 and Table S4) that is similar to a suggested potential second soluble thioesterase (PA2411) reported in the strain PAO1; this indicates a possible participation of this gene in pyoverdine synthesis in ABC1 strain. Moreover, it presents a specific acylation protein (called PvdY_II_) that produces a type II pyoverdine (Lamont et al., 2006, 2015). Additionally, *pvdE*, *pvdQ*, *pvdM*, *pvdO* and *pvdP* genes, whose products are involved in periplasmic transport of pyoverdine precursor and peptide maturation in *Pseudomonas*, were identified in the pyoverdine cluster in strain ABC1 (see Fig. 3 and Table S4). The genes coding for side‐chain modification enzymes (*pvdN* and *ptaA*) were absent in strain ABC1 (Ringel and Brüser, 2018). We also found the *fpvCDEFJK* genes, which are important for the reduction of ferripyoverdine in the periplasm and Fe^2+^ transport (Ganne *et al*., 2017; Bonneau *et al*., 2020), although the *fpvGH* genes which encode two inner membrane proteins that are part of the multiprotein complex involved in iron reduction were absent in strain ABC1 suggesting that alternative genes could replace its function.

Furthermore, the presence of PvdY_II_ and the absence of PvdF and tailoring enzymes PvdN and PtaA indicated that the siderophore produced by strain ABC1 could be similar to an azotobactin – a pyoverdine‐type siderophore produced by *A. vinelandii* (Demange *et al*., 1988). We used known Stachelhaus codes (Stachelhaus *et al*., 1999) of annotated NRPS A‐domains from *Pseudomonas* and *Azotobacter* to predict the structure. We obtained the following sequence:

Chromophore‐OHAsp‐Ala‐D‐(ᵟAc)ᵟOHOrn‐D‐Arg‐Thr‐Homoserine‐D‐Citrulline‐Citrulline‐OH‐Asp‐D‐Ser‐OHcOrn (identity of the Stachelhaus codes ≥ 90%). This sequence distinguishes it from any other known pyoverdine. Even though all pyoverdine‐related NRPS clusters of ABC1 seem to be intact, the bioinformatic approach will be corroborated in further experiments.

### Pyoverdine siderophore production

**Fig. 4:**
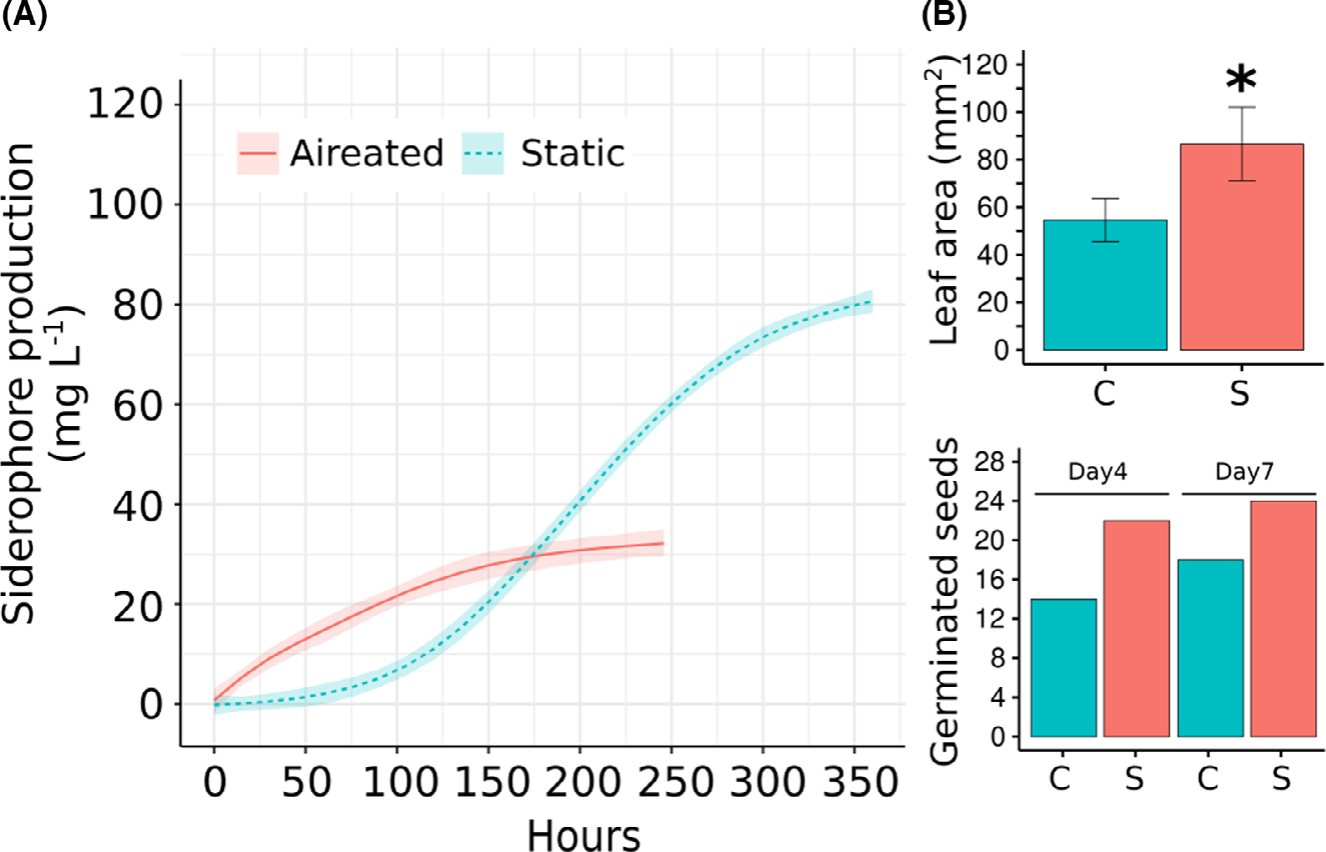
Siderophore production and plant growth promotion of *Pseudomonas* sp. ABC1. A. The *Pseudomonas* sp. ABC1 siderophore production was analysed by spectrophotometry at 400 nm from aerated and static cultures. The concentration of siderophore in mg·L^‐1^ was quantified by the FeCl_3_ titration method. B. Effect of siderophore‐rich (20 mg·l^‐1^) supernatant of ABC1 strain on *Arabidopsis thaliana* growth parameters; upper panel: leaf area of plants incubated for 15 days (*n* = 6 per treatment); C: control (distilled water); S: siderophore‐rich supernatants; and lower panel: effect of supernatants of ABC1 strain in seed germination of *A. thaliana* after 4 and 7 days of incubation (*n* = 24 per treatment). C: control (distilled water); and S: siderophore‐rich supernatant.

Our results showed an efficient production of pyoverdine by ‘*P. chilensis’* ABC1 in the AAM media in a bioreactor, with a higher production of siderophore under non‐aerated (static) conditions than under aerated condition (see Fig. 4A). The non‐aerated culture begins to produce siderophores at 72 h, reaching a maximum of 80.6 mg·l^‐1^ at 360 h, whereas the aerated culture begins to produce at 24 hours, reaching a maximum of 33.5 mg·l^‐1^ at ~ 250 h. Those production rates are similar to the values reported for other fluorescent *Pseudomonas* (Kumar *et al*., 2017). The operational conditions generate an increase in the pH from 7 to 9. Maximum production rates were reported at pH between 7 and 9 (Kumar *et al*., 2017). On evaluating the interaction of siderophore‐containing supernatants with different metal ions in solution (0.5 mM of Fe^3+^, Co^2+^, Cu^2+^ or Mn^2+^), we found that the fluorescence was quenched; this indicates that the interaction with these metals is in agreement with other pyoverdines (Ahmed and Holmström, 2014). Conversely, 0.5 mM of Zn^2+^ did not interact (see Table S2).

Several strategies must be addressed to scale‐up the bioprocess for an economical siderophore production applicable in medical, agricultural and pharmaceutical industries. Usually, glucose, sucrose, mannitol and tyrosine are used as carbon sources for the siderophore production (Kumar *et al*., 2017). Acetate is an inexpensive and sustainable substrate suited for large‐scale productions (Zhou *et al*., 2020). In *Pseudomonas*, when acetate is used as the sole carbon source, the amount of pyoverdine produced depends on the activity of glyoxylate shunt (GS; Ha *et al*., 2018). Both key enzymes of the GS – malate synthase and isocitrate lyase – were present in the genome of ABC1. In this strain, the *glcB* gene (Malate synthase) is adjacent to the *katG*. In the same context, we found the *argA* and *argE* genes that encode the enzymes involved in transformation of glutamate into ornithine, a key amino acid in the synthesis of siderophores. Further analyses are necessary to establish the mechanism of coordination in the use of acetate as carbon source for siderophore production.

### Plant growth promotion

Finally, we assessed the effect of supernatants of the strain ABC1 containing 20 mg l^‐1^ of siderophore on germination and plant growth parameters of *A. thaliana*. Supernatants did not affect the germination of seeds (see Fig. 4B); however, they induced a significant increase in foliar area (81.8 mm ± 18.1) when compared to inoculation with sterile water (52.5 mm ± 9.6; *n* = 6; two‐tailed P value of 0.0057). Siderophores have the potential of a plant growth‐promoting agent; i.e., in the vicinity of plant roots, they can limit the access to iron for pathogens (Sulochana *et al*., 2014). Several strains of fluorescent pseudomonads are known to have beneficial effects on different plant species (Passera *et al*., 2019; Rieusset *et al*., 2020). The beneficial effect of the supernatants from ABC1 strain on *A. thaliana* prompted us to propose that this novel *Pseudomonas* strain has the potential to be used as a plant growth‐promoting bacterium.

The lineage of novel ‘*P*. *chilensis*’ ABC1 and the recently established *P. kirkiae* clearly diverge from related *Pseudomonas* species regarding their metabolism, particularly in relation to iron. In this study, we discovered that these bacteria hold a high potential for being useful in a variety of biotechnological applications. These findings must be corroborated by gene synthesis and expression, metabolomic studies, and siderophore identification. In addition, the bacteria themselves may increasingly be used for biotechnological applications, in particular as a microbial inoculum in crops.

## Conflicts of interest

None declared.

## Supporting information


**Fig. S1**. Phylogenetic tree using the concatenated sequences of PCR fragments from four housekeeping genes (*16S rRNA*, *rpoE*, *rpoD*, *gyrB*) of *Pseudomonas* sp. ABC1. The total length of the concatenated sequences was 3047 bases in the final dataset. Sequences were aligned using MAFFT v7 and the ML topology shown with SH‐like approximate likelihood ratio support values (n = 1000) given at each node (values > 50% are shown) model selected was GTR + F+I + G4. The tree scale (0.1) indicates the number of nucleotide substitutions per site. Data for reference and outgroup (*Endozoicomonas elysicola*) strains were collected from the NCBI GenBank database.

**Fig. S2**. Pangenome analysis of *Pseudomonas* sp. ABC1 and related genomes. *Pseudomonas* and *Azotobacter* genomes (40) were downloaded from NCBI and analysed together using Anvio v5 (Eren et al., 2018) (Table S1). Clustering of the pangenome was generated based on the presence‐absence of genes in each genome using the mcl algorithm (inflation = 10) through Euclidean distance, and wardD2 linkage method. Heatmap represents the average nucleotide identity. Scale colour corresponds to values between 0.7‐1.

**Fig. S3**. Unique genes and functions in *Pseudomonas* sp. ABC1 based on KEGG orthologies and COG categories. A. Venn diagram of the KEGG orthologies presented in *Pseudomonas* sp. ABC1 (ABC1), *Pseudomonas kirkiae* (kirkiae), *Azotobacter* and other *Pseudomonas* genomes. B Unique COG categories in ABC1 correspond to genes that the BLASTp best hit corresponds to distant genera and/or to *Pseudomonas* with a perc. id. < 30%.

**Fig. S4**. Evolutionary relationship among FecI‐like extracytoplasmic function sigma factors (ECFsf) and their genomic contexts in *Pseudomonas* sp. ABC1. ML topology shown with SH‐like approximate likelihood ratio support values (n = 1000) given at each node (values > 50% are shown), model selected was LG + F+G4. Model selection and tree reconstruction was performed with IQ‐TREE2. Clusters of ECFsf were plotted in correspondence with their respective anti‐sigma factor genes, and numbered (1‐37) according to their location in the ABC1 genome. ECFsf related to iron acquisition from other Pseudomonas species were used as references (FoxI, PupI, FecI, FiuI, FemI, PvdS, FecI2, FpvI, HxuI, HasI, PA0149, PA2093, PA2050, PA4896), while *algU* encoding gene from *Pseudomonas* sp. ABC1 was used as an outgroup. Colour code corresponds to the different functions represented in each cluster. Ferric uptake regulator (Fur) binding sites were marked with a red arrow. The tree scale (1) indicates the number of nucleotide substitutions per site.

**Table S1**. Genomes from the genera *Pseudomonas* and *Azotobacter*.

**Table S2**. Interaction of siderophore with metal ions.

**Table S3**. Metabolic gene clusters in *Pseudomonas* sp. ABC1.

**Table S4**. Genetic analysis of *Pseudomonas* sp. ABC1.
