# Supplementary figures and images for: An unusual overrepresentation of genetic factors related to iron homeostasis in the genome of the fluorescent Pseudomonas sp. ABC1
Source: Microb Biotechnol. 2021 Jan 25;14(3):1060–72. doi: 10.1111/1751-7915.13753 (PMC8085936; doi:10.1111/1751-7915.13753)

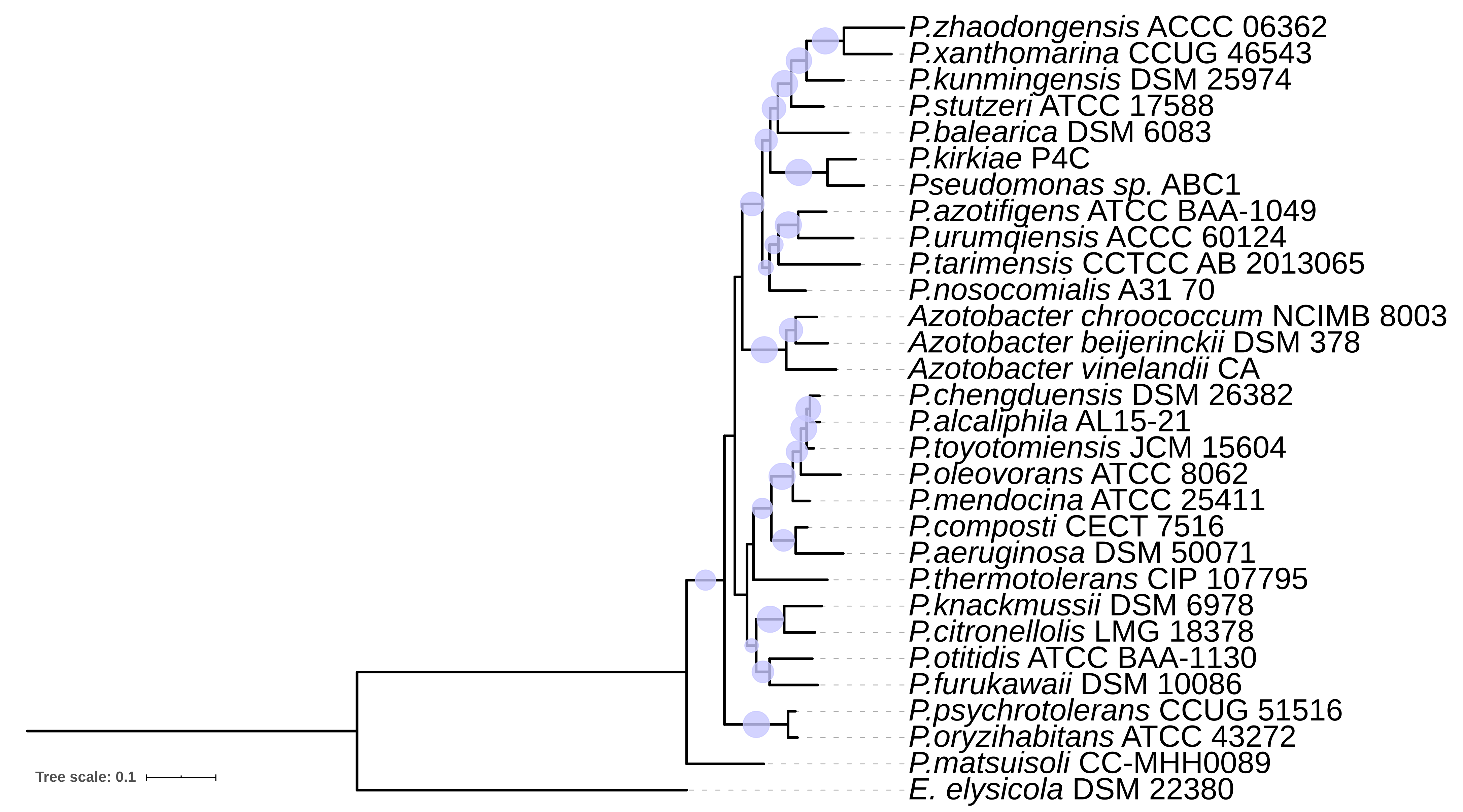

Supplement: Supplementary file 1 — Fig. S1. Phylogenetic tree using the concatenated sequences of PCR fragments from four housekeeping genes (16S rRNA, rpoE, rpoD, gyrB) of Pseudomonas sp. ABC1. The total length of the concatenated sequences was 3047 bases in the final dataset. Sequences were aligned using MAFFT v7 and the ML topology shown with SH‐like approximate likelihood ratio support values (n = 1000) given at each node (values > 50% are shown) model selected was GTR + F+I + G4. The tree scale (0.1) indicates the number of nucleotide substitutions per site. Data for reference and outgroup (Endozoicomonas elysicola) strains were collected from the NCBI GenBank database. [file MBT2-14-1060-s003.tiff]

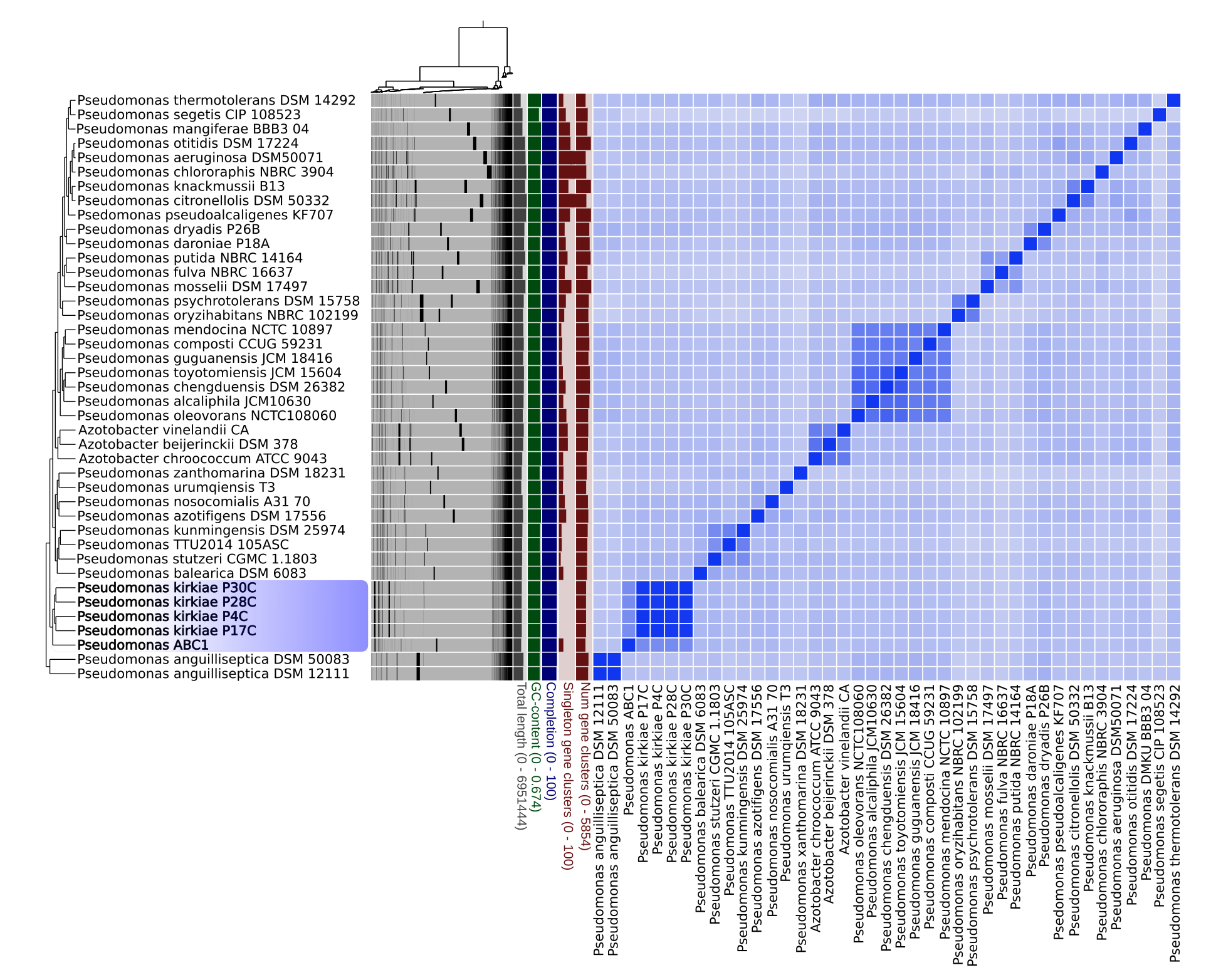

Supplement: Supplementary file 2 — Fig. S2. Pangenome analysis of Pseudomonas sp. ABC1 and related genomes. Pseudomonas and Azotobacter genomes (40) were downloaded from NCBI and analysed together using Anvio v5 (Eren et al., 2018) (Table S1). Clustering of the pangenome was generated based on the presence‐absence of genes in each genome using the mcl algorithm (inflation = 10) through Euclidean distance, and wardD2 linkage method. Heatmap represents the average nucleotide identity. Scale colour corresponds to values between 0.7‐1. [file MBT2-14-1060-s005.tiff]

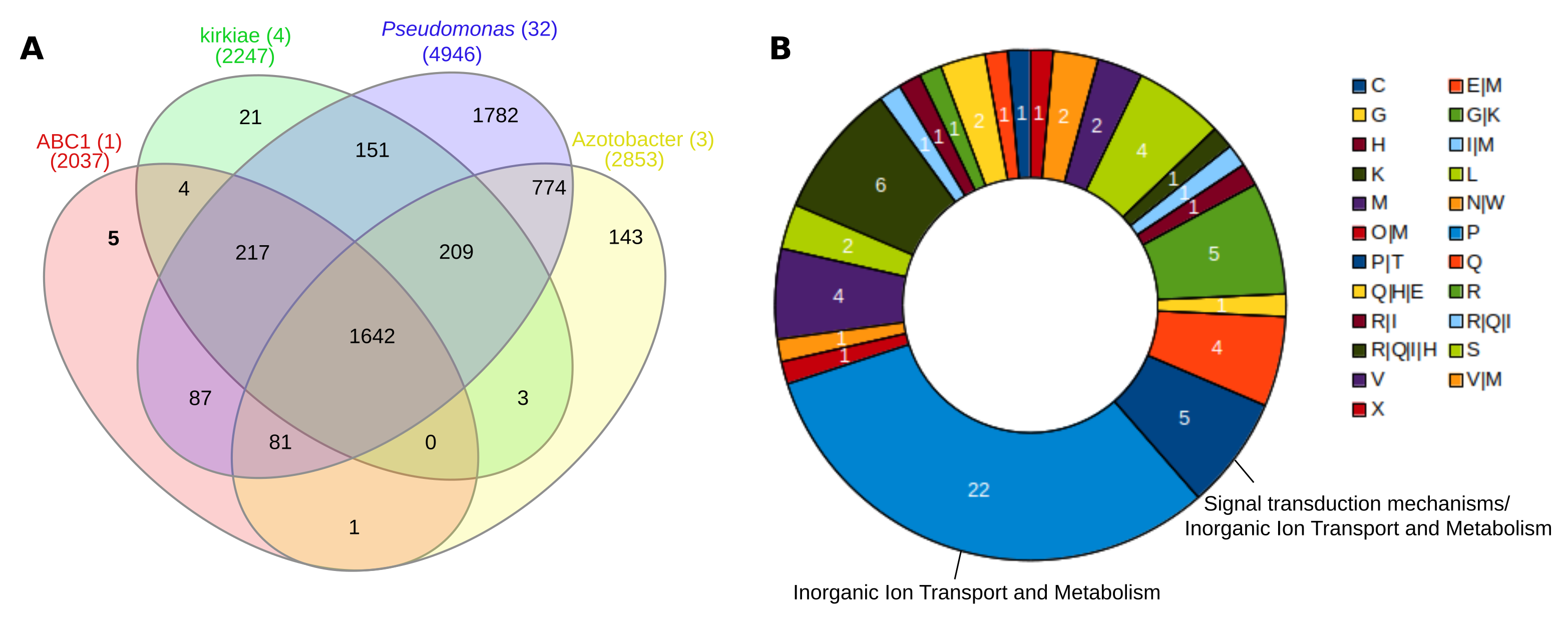

Supplement: Supplementary file 3 — Fig. S3. Unique genes and functions in Pseudomonas sp. ABC1 based on KEGG orthologies and COG categories. A. Venn diagram of the KEGG orthologies presented in Pseudomonas sp. ABC1 (ABC1), Pseudomonas kirkiae (kirkiae), Azotobacter and other Pseudomonas genomes. B Unique COG categories in ABC1 correspond to genes that the BLASTp best hit corresponds to distant genera and/or to Pseudomonas with a perc. id. < 30%. [file MBT2-14-1060-s006.tiff]

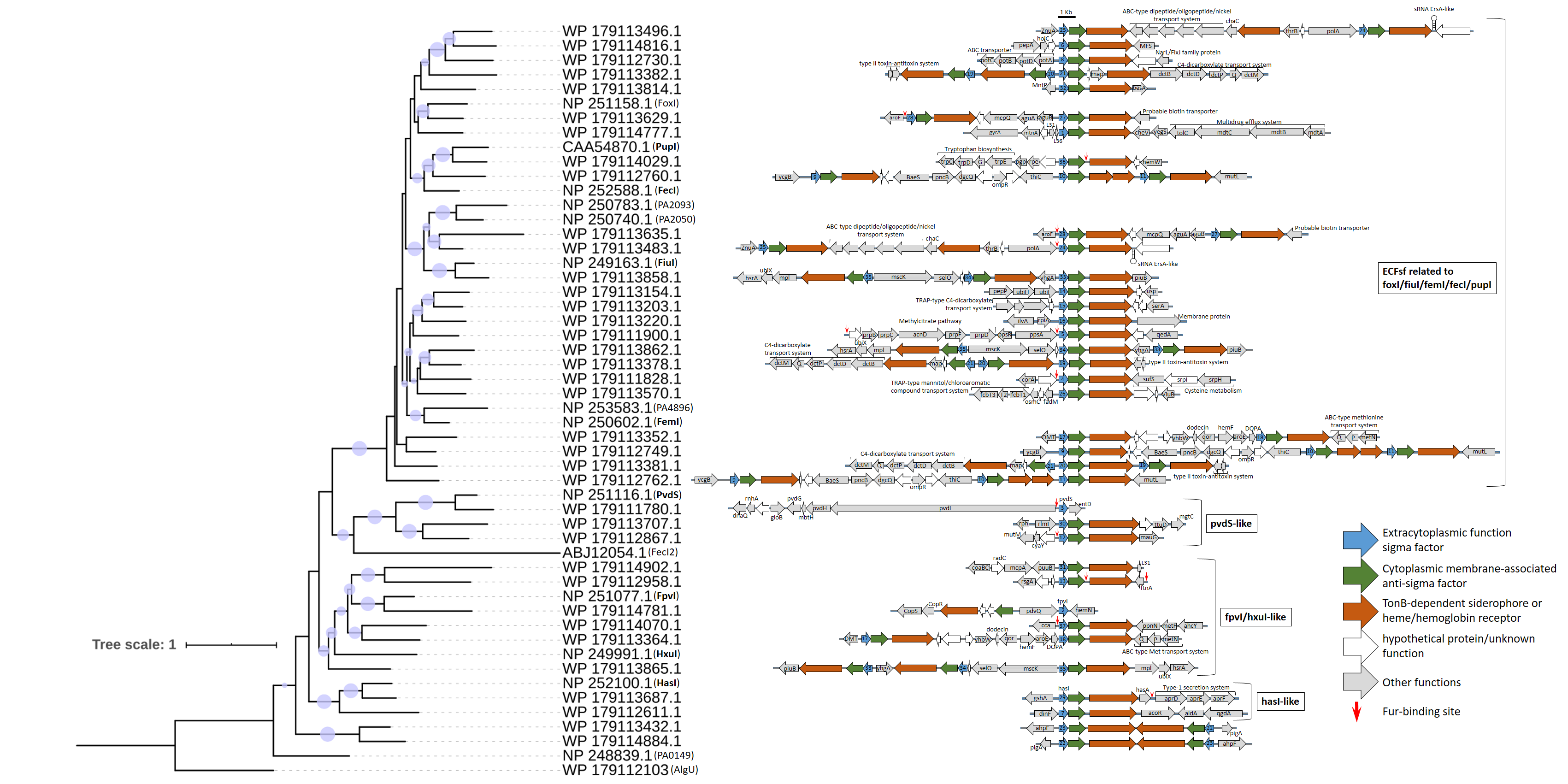

Supplement: Supplementary file 4 — Fig. S4. Evolutionary relationship among FecI‐like extracytoplasmic function sigma factors (ECFsf) and their genomic contexts in Pseudomonas sp. ABC1. ML topology shown with SH‐like approximate likelihood ratio support values (n = 1000) given at each node (values > 50% are shown), model selected was LG + F+G4. Model selection and tree reconstruction was performed with IQ‐TREE2. Clusters of ECFsf were plotted in correspondence with their respective anti‐sigma factor genes, and numbered (1‐37) according to their location in the ABC1 genome. ECFsf related to iron acquisition from other Pseudomonas species were used as references (FoxI, PupI, FecI, FiuI, FemI, PvdS, FecI2, FpvI, HxuI, HasI, PA0149, PA2093, PA2050, PA4896), while algU encoding gene from Pseudomonas sp. ABC1 was used as an outgroup. Colour code corresponds to the different functions represented in each cluster. Ferric uptake regulator (Fur) binding sites were marked with a red arrow. The tree scale (1) indicates the number of nucleotide substitutions per site. [file MBT2-14-1060-s002.tif]
